# Case Report: Multidisciplinary management of primary omental pregnancy involving intraoperative ultrasound

**DOI:** 10.3389/fmed.2025.1571248

**Published:** 2025-08-07

**Authors:** Shanshan Wang, Huaguo Shao, Yan Huang, Yanqing Li, Ting Feng, Lin Pan

**Affiliations:** ^1^Department of Ultrasound, Hangzhou Xixi Hospital Affiliated to Zhejiang Chinese Medical University, Hangzhou, China; ^2^Institute of Hepatology and Epidemiology, Hangzhou Xixi Hospital Affiliated to Zhejiang Chinese Medical University, Hangzhou, China

**Keywords:** abdominal pregnancy, primary omental pregnancy, chronic pelvic inflammation, ultrasonography, laparoscopy

## Abstract

**Introduction:**

Primary omental pregnancy (OP) is very rare, and achieving an accurate diagnosis has always been a challenge for obstetricians, gynecologists, and sonographers. Our attempt to utilize intraoperative ultrasonography has facilitated a definitive diagnosis for masses lacking obvious purple or blue characteristics under observation. This approach could provide a solution for challenges faced in intraoperative diagnosis.

**Case presentation:**

We present the case of an 18-year-old woman who was 5 months postpartum and presented with intermittent lower abdominal pain for 3 days with no obvious causes. Emergency ultrasonography diagnosed an abdominal pregnancy (AP) with abdominal and pelvic hemorrhage. Laparoscopic surgery revealed a mass on the omentum, but the surface lacked the typical purple-blue appearance. Intraoperative ultrasonography was employed, and the suspicious omental mass was shifted to the left pelvic wall. Under ultrasound guidance, the echo of a gestational sac (GS) was observed within the mass. The AP tissue and a portion of the omentum were resected. Examination of the resected omental specimen revealed chorionic villi tissue. The diagnosis was confirmed as primary OP. The operations were successful, and the patient recovered well postoperatively.

**Conclusion:**

Due to early detection, the patient received timely treatment, which played a crucial role in selecting the therapeutic plan. Chronic pelvic inflammatory disease (PID) was identified intraoperatively, suggesting that it may be a potential risk factor for OP. It is imperative for us to gain a deeper understanding of the diagnosis of primary OP, analyze its potential precipitating factors, and expand the scope of ultrasonographic examination by searching for a GS in other locations when none is visualized within the uterine cavity.

## Introduction

Under normal circumstances, the fertilized ovum implants and develops within the uterine cavity. Ectopic pregnancy (EP) refers to the implantation of the fertilized ovum outside the uterine cavity. The most common site for EP is the fallopian tubes, followed by the uterine horn, and other rare locations, including the ovary, cervix, and abdominal cavity (1). EP is a very dangerous obstetric and gynecological condition, and rupture of an EP is a leading cause of maternal mortality in early pregnancy (2).

AP is a rare form of EP, accounting for less than 1% of all EP cases. It is defined as an EP in which the gestational sac (GS) implants within the peritoneal cavity, outside the uterine cavity or fallopian tubes. Possible implantation sites include the omentum, pelvic and abdominal peritoneum, uterine surface, and abdominal organs such as the spleen, intestines, liver, and blood vessels (3, 4). In peritoneal pregnancies, fetal mortality rates range from 40 to 95%, while maternal mortality rates range from 1 to 18% (5). AP is often associated with abnormal placental implantation and inadequate blood supply, which impairs the fetus’s ability to survive (6). Due to late diagnosis, the fetus is often already deceased when peritoneal pregnancy is identified. As the gestational age increases, maternal complications can occur at any time during the prenatal, perinatal, or postnatal periods. These complications include massive hemorrhage due to spontaneous placental separation, shock, disseminated intravascular coagulation, organ failure, and death.

AP often presents with symptoms such as abdominal pain or hemorrhage, which can typically be detected through ultrasound examination. However, some cases are difficult to diagnose, and inappropriate management can lead to severe complications (7). If a diagnosis can be made early in pregnancy, there will be more time to select the appropriate treatment methods and surgical plans, significantly reducing the complications arising from EP.

## Case presentation

We present the case of an 18-year-old woman who was 5 months postpartum and admitted in November 2024 due to intermittent lower abdominal pain for 3 days. She reported her last menstrual period as October 2024, indicating a menstrual absence of 41 days. Her past menstrual cycles have been regular, with a cycle length of 30–37 days and a duration of 3–5 days. She also had a history of dysmenorrhea.

The patient had no history of long-term medication use or allergies and no prior surgeries. Both her parents, her younger brother, and her younger sister were healthy. She denied any history of genetic or familial diseases within three generations of her family, including a family history of cancer. She was pregnant and had delivered a male child via vaginal delivery 5 months ago. The patient did not take precautions with regular contraception and had no history of abortion.

Physical examination of the patient revealed a temperature of 37.1°C, a pulse rate of 119 beats per minute, a respiratory rate of 19 breaths per minute, and a blood pressure of 116/72 mmHg. Abdominal and uterine examination did not reveal any abnormal masses, tenderness, or rebound tenderness.

**Figure 1:**
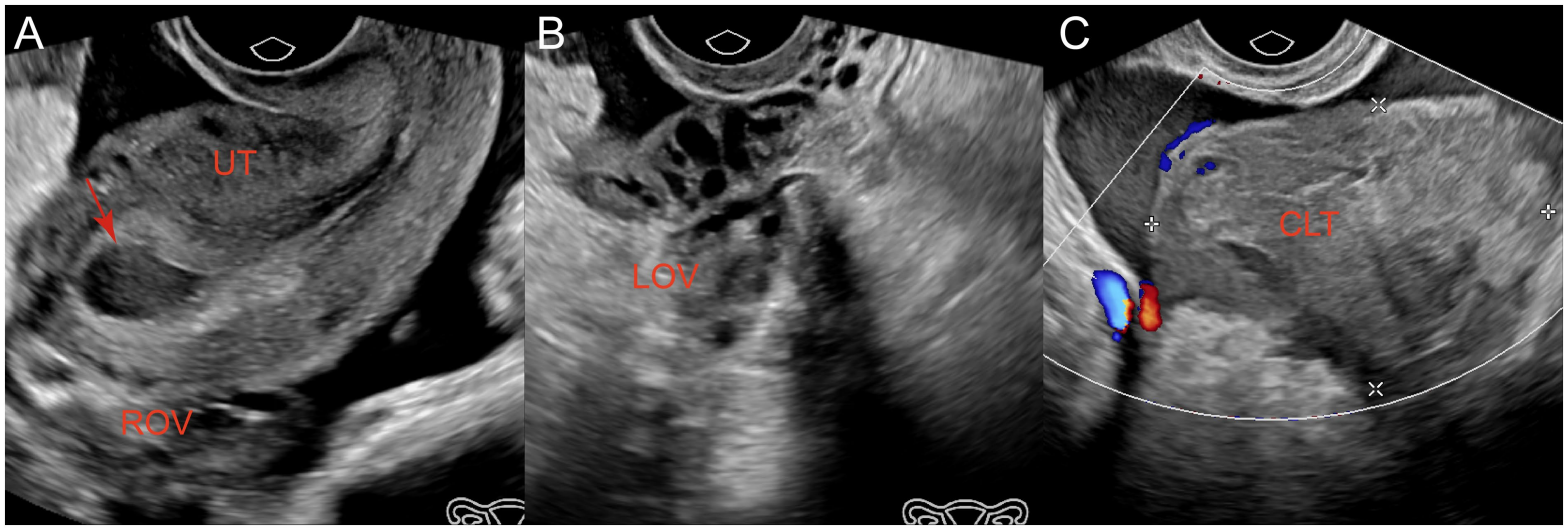
Ultrasonography revealed the following: **(A)** Uterine effusion (red arrow) and pelvic effusion and no echo of gestational sac within the uterine cavity; **(B)** No abnormalities detected in the left ovary; **(C)** A hyperechoic blood clot measuring approximately 66 mm × 47 mm within the pelvic cavity and surrounding effusion. UT, uterus; ROV, right ovary; LOV, left ovary; CLT, blood clot.

**Figure 2:**
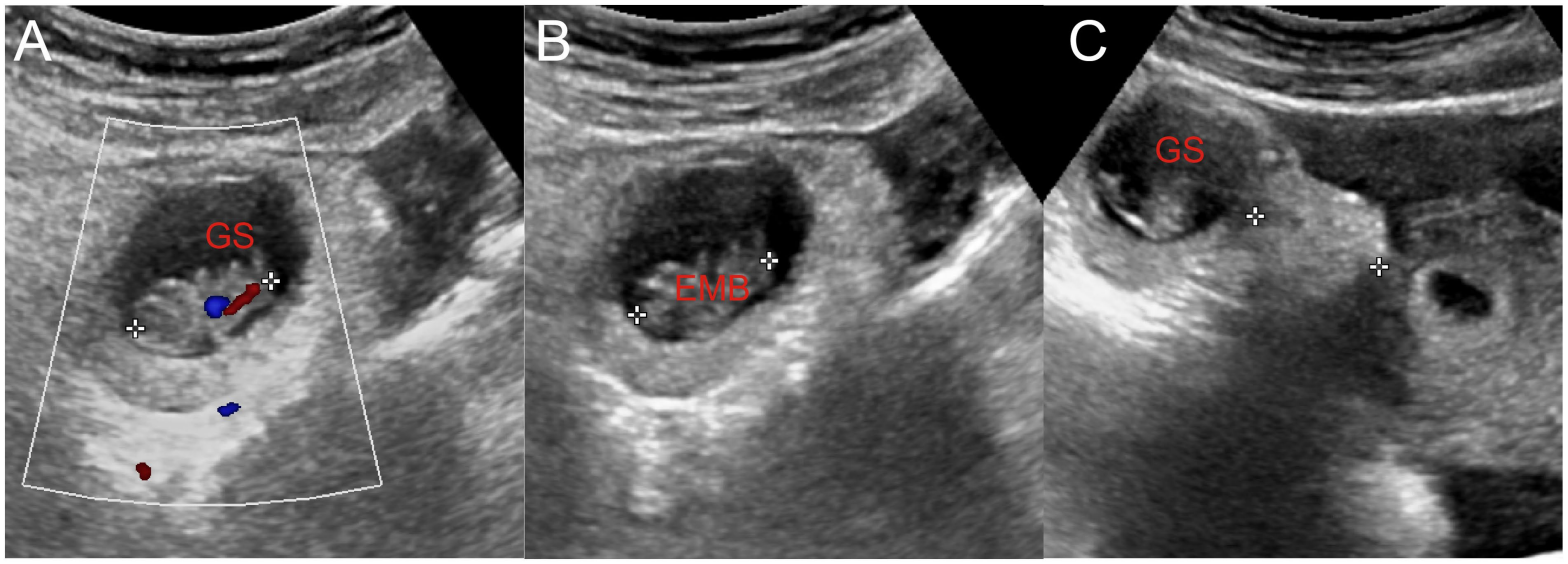
**(A)** Embryo in the gestational sac and primitive cardiac tube pulsation were observed. **(B)** The length of the embryo was approximately 2.1 cm. **(C)** Gestational sac located approximately 2.3 cm away from the uterus. GS, gestational sac; EMB, embryo.

Ultrasound showed no GS within the uterine cavity. A GS, measuring approximately 29 mm × 25 mm × 26 mm, was observed 23 mm above and slightly to the left of the uterus. The GS contained an echo of an embryo approximately 21 mm in length, suggesting a gestation of more than 8 weeks with a detectable fetal heartbeat. The ultrasound diagnosis was AP, pelvic and abdominal cavity effusion, pelvic hematoma formation, and uterine cavity effusion (Figures 1, 2). On the day of admission, blood tests revealed a human chorionic gonadotropin (hCG) level of 69,305 mIU/L. Blood routine tests indicated a hemoglobin level of 110 g/L, a white blood cell count of 14.67 × 10^9^/L, and a rapid C-reactive protein level of < 0.5 mg/L.

The primary diagnosis was EP with intra-abdominal hemorrhage. Abdominal pain needed to be differentiated from ovarian corpus luteum rupture and pelvic inflammatory disease (PID). Based on ultrasound findings and test results, these conditions were temporarily not considered. The patient’s current diagnosis of EP was clear.

Based on the ultrasound findings of AP, pelvic and abdominal cavity effusion, and pelvic hematoma, the patient and her family consented to laparoscopic exploration, with the preparation for salpingectomy on the affected side or AP tissue removal. They were informed of the potential risks and complications of the surgery. The patient and her family expressed informed consent by signing the consent form. Emergency laparoscopic surgical exploration was performed on the same day.

**Figure 3:**
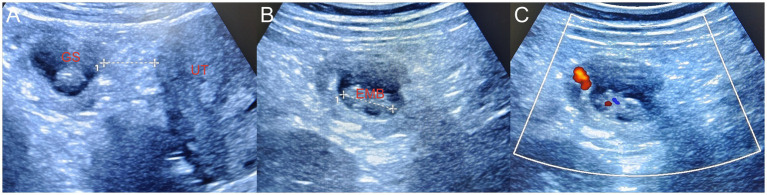
**(A)** During laparoscopy surgery, the suspicious mass on the omentum was flipped to the left pelvic wall. Bedside ultrasonography revealed a primary omentum pregnancy, and the gestational sac was located approximately 2.5 cm away from the uterus. **(B)** The length of the embryo was approximately 2.1 cm. **(C)** CDFI demonstrated the primitive cardiac tube pulsation of the embryo. Since the abdominal and pelvic hemorrhage had been cleared during the surgery, no surrounding effusion was displayed. GS, gestational sac; EMB, embryo; UT, uterus, CDFI, color Doppler flow imaging.

**Figure 4:**
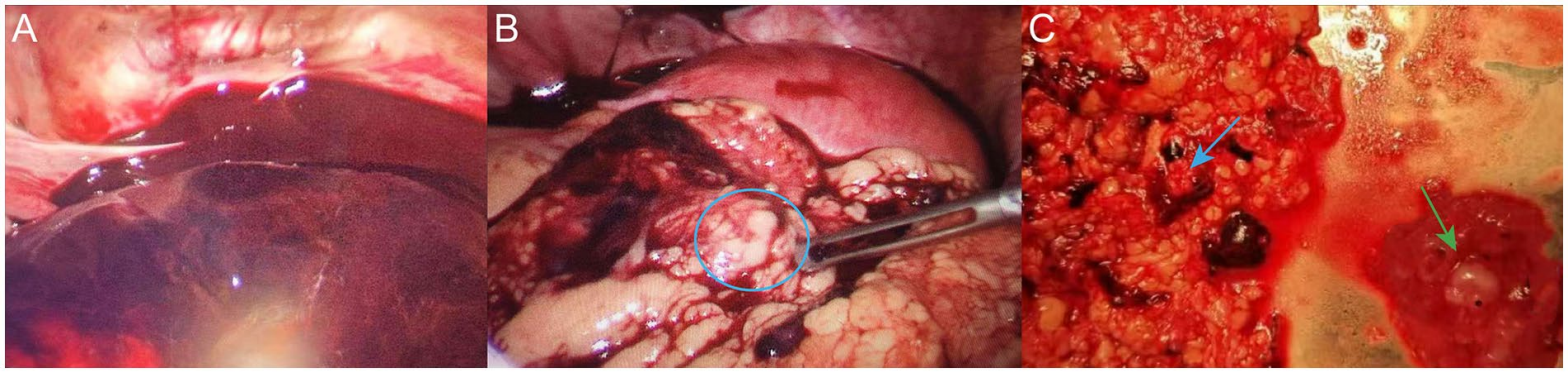
**(A)** Dark red blood and blood clots were observed in the abdominal and pelvic cavities intraoperatively. **(B)** Embryonic tissue was observed on the omentum (blue circle). **(C)** Resected embryonic tissue (green arrow) and partial omentum tissue (blue arrow).

Intraoperatively, approximately 800 mL of dark red blood and blood clots were found in the abdominal and pelvic cavity. The uterine surface was smooth, with adhesion bands formed between the posterior uterine wall and the pelvic floor and partial adhesion of the intestine to the pelvic wall. The bilateral fallopian tubes and ovaries appeared normal with no visible ruptures, and no tissue obstruction was observed at the fimbriated ends of the bilateral fallopian tubes. A 50 mm × 40 mm mass was found on the omentum above the uterus, with no purple-blue appearance on the surface. An intraoperative consultation with the ultrasound department was requested. Ultrasound with a disposable sterile probe cover revealed no GS within the uterus. The suspicious omentum mass was moved toward the left pelvic wall, and ultrasound guidance revealed a GS with an echo of an embryo and fetal heartbeat at the left pelvic wall mass, confirming an OP (Figure 3). An abdominal surgeon was asked to assist in the surgery, and the intra-AP tissue and a portion of the omentum were resected. On examination of the resected omentum specimen, obvious chorionic villi tissue was visible (Figure 4). The postoperative diagnosis was OP with intra-abdominal hemorrhage. The surgery was successful, and the patient was safely taken back to the ward with no bleeding from the incision.

Postoperatively, the patient received intravenous infusions of 100 mL sodium chloride solution and 1.5 g cefuroxime twice daily for 48 h to prevent infection. On the first postoperative day, blood tests revealed a white blood cell count of 14.11 × 10^9^/L, a hemoglobin level of 97 g/L, a rapid C-reactive protein level of 14.57 mg/L, and a serum amyloid A level of 86.05 mg/L. The low hemoglobin level was consistent with blood loss anemia, and intravenous iron sucrose was administered to treat the anemia. The slightly elevated blood indices and body temperature were due to postoperative stress reactions. The patient had no symptoms. The blood hCG level was 21,046 mIU/ml, with a decrease of more than 50% compared to the preoperative level, consistent with postoperative changes. On the fourth postoperative day, a follow-up blood test showed an hCG level of 2,733 mIU/ml and a hemoglobin level of 100 g/L. The pathology report confirmed an OP.

The patient recovered well with good wound healing and a significant decrease in blood hCG levels. She was discharged from the hospital and instructed to return for a follow-up serum hCG and blood examination 1 week later. The patient was advised to increase iron-rich foods in her diet, and return for a follow-up visit promptly if she had vaginal bleeding.

After discharge, the patient returned for follow-up visits on 21 November 2024 and 30 November 2024, with blood hCG levels of 286 mIU/ml and 71.8 mIU/ml, respectively. The patient had no discomfort and continued to be monitored during follow-ups for blood hCG levels until they were negative.

The patient provided written informed consent for their personal and clinical details, including any images, to be published in this study.

## Discussion

AP is an extremely rare form of EP. Due to its rarity, the exact incidence of AP is inestimable, with reports suggesting an incidence ranging from approximately 1 in 10,000 to 1 in 30,000 pregnancies (8). Among all EPs, the incidence of AP is less than 1%, but its mortality rate is significantly higher than that of tubal pregnancy (3). This is because the blastocyst can invade maternal organs and blood vessels, leading to hemorrhage or rupture of the maternal organs. It is possible for fetuses of AP to reach term and survive up to the perinatal period; however, these are extremely rare occurrences (9). AP can be classified into primary and secondary forms. Primary AP occurs when the fertilized ovum implants directly into the abdominal cavity. Secondary AP often arises following the abortion or rupture of a tubal pregnancy or after the rupture of an ovarian pregnancy with subsequent implantation in the abdominal cavity. Primary AP is even more rare and is defined by Studdiford’s three criteria, which are as follows: (1) normal fallopian tubes with no evidence of recent or remote trauma, the absence of any uteroperitoneal fistula, and the presence of a pregnancy related exclusively to the peritoneal surface and early enough to eliminate the possibility of secondary implantation after a primary nidation within the tube. In our patient, the pregnancy was in its early stages, and examination of the omentum specimen resected during surgery revealed chorionic villi tissue. Pathological results were consistent with OP, and based on intraoperative findings, we classified it as a primary OP. The implantation site of AP can be anywhere in the abdominal cavity. In this patient, the fertilized ovum implanted in the omentum, which is referred to as OP and is a subclass of AP.

The risk factors for AP remain unclear. Studdiford outlined various risk factors that lead to AP, including PID, smoking, tubal surgery, previous endometriosis, and assisted reproductive technology. However, only 50% of the reported cases involve women with identified risk factors associated with AP (4, 10). In this patient, both pregnancies were natural conceptions without assisted reproductive technology. Intraoperative findings revealed adhesive bands between the posterior wall of the uterus and the pelvic floor, as well as between part of the intestine and the pelvic wall, suggesting chronic PID. No signs of endometriosis were found during the surgery. PID alters the microenvironment of the fallopian tubes and pelvic cavity, interfering with the normal transport of the fertilized ovum, which may then deviate from its normal implantation pathway and implant in the omentum. Regarding chronic PID, ultrasound can observe effusion in the pelvic cavity and morphological abnormalities of the fallopian tubes, such as thickening and bending, or may reveal tissue adhesions within the pelvic cavity. Genital gonococcal infection triggers a cascade of reactions involving both humoral and cellular immune mechanisms, leading to tissue damage and adverse reproductive outcomes. It is estimated that approximately 10% of chlamydial cervical infections ascend to the upper genital tract and cause pelvic inflammatory disease (PID), although this proportion is less well-defined compared to that of gonococcal infection. Regrettably, the patient did not undergo pathogen detection related to PID after the surgery (11). In this case, preoperative ultrasound did not reveal sonographic changes indicative of PID. This could be due to the influence of intraperitoneal hemorrhage on the interpretation of the ultrasound or because the adhesions were in areas not easily observed by ultrasound, such as between the posterior wall of the uterus and pelvic floor, as well as part of the intestine and pelvic wall. Medical treatment is an effective therapeutic option for early unruptured ectopic pregnancies. However, surgical intervention is indicated for symptomatic patients who fail medical treatment. Given that the patient experienced rupture of ectopic pregnancy with significant bleeding, emergency laparoscopic surgery was chosen (12).

There is limited research on the management of AP. A review of 17 cases by Chen et al. revealed that the preoperative diagnostic rate of AP remains low, with only 29.41% of cases being diagnosed preoperatively (13). Confirming the diagnosis in early pregnancy remains challenging due to the similarity in clinical symptoms between AP and EP, particularly when the mass is adjacent to the adnexa area. This case is unique because we diagnosed AP preoperatively around 8 weeks of gestation. During transvaginal sonography, the sonographer carefully examined the uterine cavity and bilateral adnexa but did not find a GS or EP mass. Due to the discrepancy with clinical features and blood hCG levels, a comprehensive transabdominal sonography with an expanded scanning range was performed. The scan revealed a GS in the abdominal cavity, 23 mm above and slightly to the left of the uterus. This experience serves as a valuable reminder that, when patients have high blood hCG levels but no evidence of intrauterine pregnancy or common EP, the possibility of AP should be considered, and the examination scope should be immediately expanded to avoid missing the diagnosis. During the operation, intraoperative ultrasound was instrumental in characterizing the mass, which did not exhibit the typical purple-blue appearance of EP seen during laparoscopy. Since laparoscopy required insufflation, which hindered direct ultrasound observation, the suspicious omentum was moved to the left pelvic wall. This move facilitated both ultrasound examination and confirmation that the GS was located there, providing a basis for diagnosis. This case also demonstrates that ultrasound diagnosis of early AP is feasible and worth promoting. However, some APs in special locations may still rely on magnetic resonance imaging (MRI) or intraoperative findings.

## Conclusion

AP is rare, with diverse and non-specific clinical symptoms, making preoperative diagnosis challenging. However, we can still strive to diagnose AP by expanding the scope of examination. Abdominal ultrasonography plays a crucial role in the diagnosis and localization of AP. Laparoscopy is the primary treatment for AP, especially when the patient is hemodynamically stable. The multidisciplinary approach we employed in this case, which included intraoperative ultrasonography, may provide some insights for addressing similar cases in the future.

## Data Availability

The original contributions presented in the study are included in the article/supplementary material; further inquiries can be directed to the corresponding author.
